# Opinion article: Neurosurgical treatment for neuro-ophthalmologic conditions

**DOI:** 10.3389/fopht.2023.1189725

**Published:** 2023-04-20

**Authors:** Zoe R. Williams, Andrew G. Lee, Clare L. Fraser, Julie Falardeau, Prem S. Subramanian, John J. Chen, Bayan Al Othman, Marc J. Dinkin, Susan P. Mollan, Nagham Al-Zubidi, Edward Vates

**Affiliations:** ^1^ Department of Neurosurgery, University of Rochester Medical Center, NY, Rochester, United States; ^2^ Department of Ophthalmology, Houston Methodist Hospital, TX, Houston, United States; ^3^ Blanton Eye Institute, Houston Methodist Hospital, TX, Houston, United States; ^4^ Save Sight Institute, Faculty of Medicine and Health, The University of Sydney, Sydney, Australia; ^5^ Department of Ophthalmology, Oregon Health and Science University, OR, Portland, United States; ^6^ Department of Ophthalmology, UCHealth Sue Anschutz Rodgers Eye Center, CO, Denver, United States; ^7^ Department of Ophthalmology, Mayo Clinic, MN, Rochester, United States; ^8^ Department of Ophthalmology, University of Rochester Medical Center, NY, Rochester, United States; ^9^ Weill Cornell Medical Center, New York-Presbyterian, NY, New York, United States; ^10^ Department of Ophthalmology, University Hospitals Birmingham NHS Foundation Trust, Birmingham, United Kingdom; ^11^ Department of Head and Neck Surgery, University of Texas MD Anderson Cancer Center, TX, Houston, United States

**Keywords:** neurosurgery, neuro-ophthalmology, idiopathic intracranial hypertension, ophthalmic artery aneurysms, sellar tumors, optic pathway tumors, intracranial pressure disorders, venous sinus stenting

## Abstract

A significant role of the neuro-ophthalmologist is to counsel patients on appropriate management and anticipated visual prognosis for conditions affecting the afferent and efferent visual systems, including those requiring neurosurgical treatment. However, the literature regarding anticipated neuro- ophthalmologic prognosis after neurosurgical intervention for cerebral aneurysms, sellar lesions, optic pathway tumors, and elevated intracranial pressure is limited with many key questions unanswered. For example, if a cerebral aneurysm is equally amenable to clipping or endovascular coiling, is there a preferred approach in terms of visual prognosis based on aneurysm location? Is dural venous sinus stenting (VSS) for idiopathic intracranial hypertension (IIH) superior, equivalent or inferior to shunting in terms of visual recovery and safety profile? Landmark studies on pituitary tumors using pre-operative optical coherence tomography (OCT) imaging of the optic nerve head to predict visual recovery after surgical decompression of the optic chiasm have changed neuro-ophthalmologic practice and enabled patients to be better informed regarding expected visual outcomes. 1,2 In order to optimize an interdisciplinary team approach to patient care, further studies of visual outcomes for neuro- ophthalmologic conditions requiring neurosurgical intervention are needed.

This research topic series highlights current studies of visual outcomes of neuro-ophthalmologic conditions related to cerebral aneurysms, sellar and optic pathway tumors, and intracranial pressure disorders.

Surgical clipping of ophthalmic artery aneurysms can be technically difficult and carries a significant risk of vision loss; therefore, an endovascular approach may be used for certain anatomic configurations despite higher aneurysm recurrence rates.3 Determining the safety of a curative endovascular approach to ophthalmic artery aneurysms can be aided by intraoperative indirect fundoscopy during balloon test occlusion of the ophthalmic artery (https://www.frontiersin.org/articles/10.3389/fopht.2022.940479). Indirect fundoscopy has the advantage of requiring minimal intraoperative time without need to awaken the patient. Furst et al. describe a case of an ophthalmic aneurysm and superior hypophyseal artery which were successfully occluded by endovascular flow diversion and adjunctive coiling after confirmation of choroidal blush from external carotid artery collateral circulation *via* angiography and preserved retinal perfusion *via* indirect fundoscopy during balloon test occlusion (https://www.frontiersin.org/articles/10.3389/fopht.2022.940479). It is important to establish whether there is a distal collateral circulation as this will decrease the pressure gradient across the stent, enabling thrombus formation within the aneurysm and endothelization of the stent (https://www.frontiersin.org/articles/10.3389/fopht.2022.940479).

Neuro-ophthalmologic evaluation is essential in the management of sellar lesions for diagnosis, baseline assessment of afferent or efferent visual dysfunction and to guide visual prognosis.1,2 Al-Bader et al. present a comprehensive review of OCT imaging and visual field testing in the evaluation of sellar lesions, radiologic characteristics of various sellar lesions and therapeutic options https://www.frontiersin.org/articles/10.3389/fopht.2022.970580). This study discusses the range of visual field defects seen with sellar lesions, including monocular visual field defects seen in up to one third of cases (https://www.frontiersin.org/articles/10.3389/fopht.2022.970580). In addition, it highlights that preservation of ganglion cell complex on pre-operative OCT may be more predictive of visual recovery with surgical decompression than preserved peripapillary retinal nerve fiber layer thickness (https://www.frontiersin.org/articles/10.3389/fopht.2022.970580). Visual recovery with decompressive surgery has been attributed to a combination of restoration of axonal conduction accounting for immediate improvement, and improved axoplasmic flow and remyelination accounting for delayed improvement (https://www.frontiersin.org/articles/10.3389/fopht.2022.970580).

Maheshwari et al. provide a thorough overview of the neuro-ophthalmologic presentation of optic pathway gliomas and indications for surgery, chemotherapy, radiation, radiosurgery, and immunotherapy (https://www.frontiersin.org/articles/10.3389/fopht.2022.992673). The appropriate management of optic pathway gliomas is individualized (https://www.frontiersin.org/articles/10.3389/fopht.2022.992673). Frequently, optic pathway gliomas are initially observed for stability and in the setting of neurofibromatosis type I, there is a usually a more favorable visual prognosis (https://www.frontiersin.org/articles/10.3389/fopht.2022.992673). Chemotherapy is often considered first in patients who are symptomatic with progressive vision loss (https://www.frontiersin.org/articles/10.3389/fopht.2022.992673). Radiation therapy may be considered in patients who fail medical therapy but its use is limited in children due to risk of endocrinologic side effects or cognitive impairment (https://www.frontiersin.org/articles/10.3389/fopht.2022.992673). Surgery is generally reserved for exophytic lesions or obstructive hydrocephalus (https://www.frontiersin.org/articles/10.3389/fopht.2022.992673). Immunotherapy is a targeted therapy for certain pediatric low grade gliomas expressing specific mutations (https://www.frontiersin.org/articles/10.3389/fopht.2022.992673). Maheshwari et al. recommend consideration of tumor biopsy to enable targeted therapies with MEK or BRAF inhibitors (https://www.frontiersin.org/articles/10.3389/fopht.2022.992673).

IIH can be a potentially blinding disease without timely intervention but the literature leaves many unanswered questions regarding optimal management. 4-7 Miri et al. propose a “fast-tracking” strategy for multidisciplinary evaluation and management of vision-threatening IIH to optimize patient outcome and decrease length of hospital stay (https://doi.org/10.3389/fopht.2022.923092). The interdepartmental team should include Neuro-ophthalmology, Neurology, Neurosurgery, and Neuroradiology/Interventional Radiology (https://doi.org/10.3389/fopht.2022.923092). Neuro- ophthalmologic assessment with formal visual field testing and OCT imaging is performed to assess risk of vision loss due to papilledema. Urgent neuroimaging (either MRI-MRV brain or CT-CTV head) to rule out a structural etiology is followed by lumbar puncture to rule out secondary causes of elevated intracranial pressure (https://doi.org/10.3389/fopht.2022.923092). Surgical options for vision- threatening IIH include shunting procedures for cerebrospinal fluid (CSF) diversion, VSS to reduce cerebral venous hypertension and enable CSF reabsorption, and optic nerve sheath fenestration (ONSF) to enable CSF release and decompression at the optic nerve level. Admission for lumbar drain is recommended for patients with fulminant IIH or impending vision loss with inability to tolerate/lack of response to maximal medical therapy in whom CSF diversion *via* shunt, cerebral dural VSS or ONSF cannot be performed within 24 hours (https://doi.org/10.3389/fopht.2022.923092).

Cerebral VSS has been increasingly employed to treat medically refractory IIH over the past 15 years.4,7 In this collection, Reid et al. report a retrospective cohort study including 32 patients with medically refractory IIH (18% of 226 patients in the study), 80% of whom underwent transverse VSS based on significant pressure gradient on manometry (https://www.frontiersin.org/articles/10.3389/fopht.2022.885583). Papilledema resolved in 96% of patients undergoing transverse VSS with a statistically significant improvement of perimetric mean deviation in 98% of eyes and only 6% patients requiring an additional procedure (https://www.frontiersin.org/articles/10.3389/fopht.2022.885583). Over 80% of patients were able to discontinue acetazolamide (https://www.frontiersin.org/articles/10.3389/fopht.2022.885583). Two patients developed restenosis adjacent to the stent, with one of the two cases requiring a second stent. One patient did not have resolution of increased superior sagittal sinus pressure from stenting despite resolution of pressure gradient across the transverse sinus stenosis and required subsequent shunting (https://www.frontiersin.org/articles/10.3389/fopht.2022.885583). The only complication of stenting seen in this cohort was ingress of an arachnoid granulation through the stent mesh, which did not require intervention (https://www.frontiersin.org/articles/10.3389/fopht.2022.885583). Almost all patients developed headaches after stenting which were presumed to be secondary to dural stretch and spontaneously resolved within a few weeks (https://www.frontiersin.org/articles/10.3389/fopht.2022.885583).

Another retrospective review of 86 patients with IIH undergoing VSS at two academic medical centers between 2015-2021 showed significant reduction in papilledema postoperatively, while 77% had improvement of headache and 67% had resolution of pulsatile tinnitus (https://www.frontiersin.org/articles/10.3389/fopht.2022.910524). Interestingly, this study showed that only 32% no longer required intracranial pressure lowering medication 3 months post-operatively and that nearly one-quarter needed more than one medication (https://www.frontiersin.org/articles/10.3389/fopht.2022.910524). This study also reported an approximate 6% complication rate from stenting including 2 patients with cerebral hemorrhage, one with post-operative seizures, one with femoral hematoma and one with severe post-operative pain (https://www.frontiersin.org/articles/10.3389/fopht.2022.910524). This literature is nicely summarized in the editorial by Dinkin et al. which emphasizes the need for multicenter randomized controlled clinical trials to determine the efficacy of VSS in vision threatening or medically refractory IIH and benefits of a “fast track” treatment strategy (https://www.frontiersin.org/articles/10.3389/fopht.2022.1021667).

## Author contributions

ZW wrote the individual article summaries and drafted the manuscript. ZW, AL, CF, JF, PS, JC, BA, MD, SM, NA-Z, and EV reviewed and edited the manuscript and approved the final version for publication. All authors contributed to the article and approved the submitted version. 

